# Morphology Control of Energy-Gap-Engineered Nb_2_O_5_ Nanowires and the Regioselective Growth of CdS for Efficient Carrier Transfer Across an Oxide-Sulphide Nanointerface

**DOI:** 10.1038/s41598-017-05292-2

**Published:** 2017-07-07

**Authors:** Tomoki Shinohara, Miyu Yamada, Yuki Sato, Shohei Okuyama, Tatsuto Yui, Masayuki Yagi, Kenji Saito

**Affiliations:** Department of Materials Science and Technology, Faculty of Engineering, Niigata University, 8050 Ikarashi-2, Niigata, 950-2181 Japan

## Abstract

Semiconductor nanowires with both nano- and micrometre dimensions have been used as effective materials for artificial photosynthesis; however, a single synthesis approach to provide rational control over the macroscopic morphology, which can allow for the high-throughput screening of photocatalytic performance, and carrier transfer between oxide and sulphide nanostructures has been poorly known. Our recent findings indicate that a single parameter, Nb foil thickness, in a vapor-phase synthesis method can alter the macroscopic morphology of resulting Nb_2_O_5_ nanowires. Thick Nb foil results in a free-standing Nb_2_O_5_ film, whereas a thinner foil leads to fragmentation to give a powder. During the synthesis process, a Rh dopant was provided through metal-organic chemical vapor deposition to reduce the Nb_2_O_5_ energy gap. Upon irradiation with visible light (*λ* > 440 nm), the free-standing nanowire film [Nb_2_O_5_:Rh-NW(F)] showed photoanodic current with a Faradaic efficiency of 99% for O_2_ evolution. Under identical irradiation conditions, the powdered counterpart [Nb_2_O_5_:Rh-NW(P)] showed activity for O_2_ evolution in the presence of an electron acceptor. The poor water-reduction ability was greatly enhanced by the Au-catalysed vapor-liquid-solid (VLS) growth of H_2_-evolving CdS onto the reduction sites of Nb_2_O_5_:Rh-NW(P) [Au/CdS/Nb_2_O_5_:Rh-NW(P)].

## Introduction

Artificial photosynthesis harnessing sunlight to construct chemical bonds for use as a fuel is a key technologies to move towards a sustainable society^1–3^. Among the relevant studies, one-dimensional nanostructuring of a semiconductor photocatalyst has a great potential to accelerate the development of this technology, which has been the focus of several reports^4–6^. The unique photoresponse of nanowires results from their inherent physical properties, including very high aspect ratio and large surface area. However, the high aspect ratio imposes a limitation to the macroscopic morphology, *i.e*., the nanowires must align at a right angle to the planar substrate for use in photoelectrocatalysis, to weaken the light scattering and to streamline carrier transfer between the nanowire and substrate. In general, synthetic routes entirely from solution for photoelectrocatalyst materials for water splitting are still limited, and previously, the syntheses of aligned nanowires as photoelectrocatalysts and non-oriented nanowires as heterogeneous photocatalysts have been rooted in well-established, vapor- and liquid-phase methods, respectively^7–14^.

Niobium pentoxide with a composition formula of Nb_2_O_5_ is a white semiconductor material with both favourable photocatalytic performance and high chemical stability. The photocatalytic applications of Nb_2_O_5_, particularly regarding its visible-light response, are continuously widening. Huang, H. *et al*. reported that mesoporous, nitrogen-doped Nb_2_O_5_ showed an efficient visible-light performance for H_2_ evolution^15^. Bonding Nb_2_O_5_ with another semiconductor material, such as graphitic carbon nitride (C_3_N_4_)^16^, reduced graphene oxide^17^, or cadmium sulphide (CdS)/nitrogen-doped graphene^18^, opens ways to harness visible light as well. However, even though the valence-band maximum of Nb_2_O_5_ is high enough to oxidize water (ca. 3 eV vs. NHE), reports on the O_2_ evolution reaction over Nb_2_O_5_ have been rare, regardless of the exploitable wavelength of light^19^.

Contrary to a single photocatalyst, the combination of two separate photocatalysts with H_2_ and O_2_ evolution centres, referred to as a Z-scheme photocatalyst, liberalizes the thermodynamic restrictions on water splitting, where the water-reduction/oxidation potentials must be located between the potential energies of the conduction-band minimum and valence-band maximum of the photocatalysts^20^. Hence, many combinations have been screened and subsequently discovered^1, 2^. Among them, oxide-sulphide combinations are promising candidates because oxides and sulphides possess strong oxidation and reduction capabilities, respectively^21–31^. Ideally, an H_2_-evolving sulphide should be placed on the reduction site of the oxide because this positional relationship allows for efficient carrier transfer, and excessive sulphide utilization or deposition on the oxide could provoke a light-shielding effect. However, such a strategy is poorly understood.

A vapor-liquid-solid (VLS) mechanism is an established technology that allows for controlled growth of nanostructured material by a metal catalyst with control of the location, size, etc.^32, 33^. Of course, this technology can be applied to metal-sulphide deposition onto an oxide photocatalyst to construct a Z-scheme photocatalyst. However, the extremely low permeability of the vapor source into the solid hampers the application of the VLS mechanism to powdered photocatalysts. On the other hand, photodeposition is a well-known technique in which a metal cocatalyst is formed on the reduction site of the photocatalyst^19^. Therefore, a combination of VLS and photodeposition could pave a new path to construct oxide-sulphide composite photocatalysts if the permeability problem is solved adequately.

**Figure 1:**
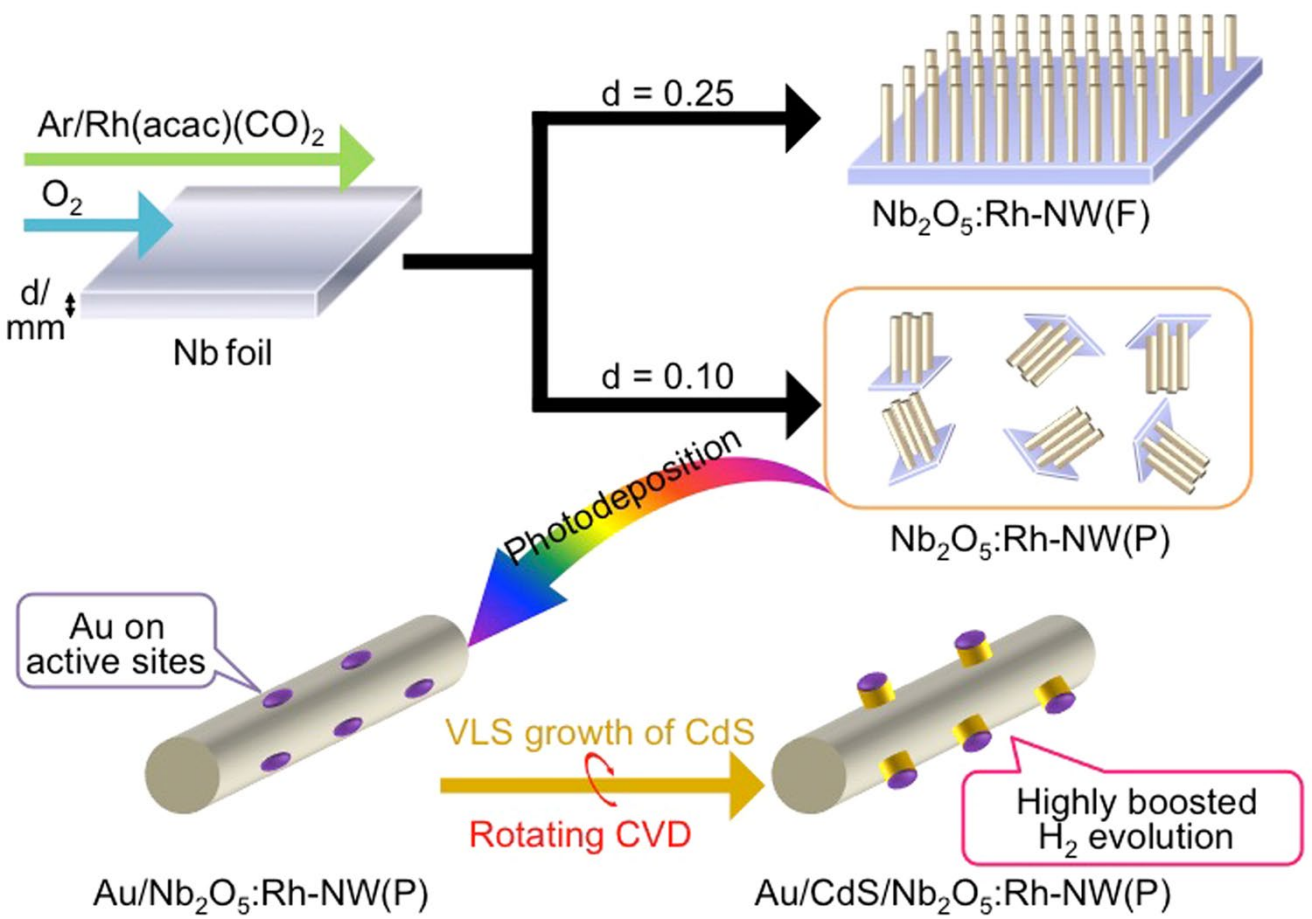
An overview of the morphology control and the rational 3D functionalization of the Rh-doped Nb_2_O_5_ nanowire.

Here, we report a single synthesis approach of band-gap-engineered Nb_2_O_5_ nanowires with two different macroscopic morphologies, namely, a free-standing film and a powdered form. In addition, the physical rotation^34^, used in the VLS growth of CdS on the reduction site of the nanowire powder, was found to have an obvious effect on the H_2_ evolution activity (Fig. 1).

## Results and Discussion

### Structure characterization and physical properties

**Figure 2:**
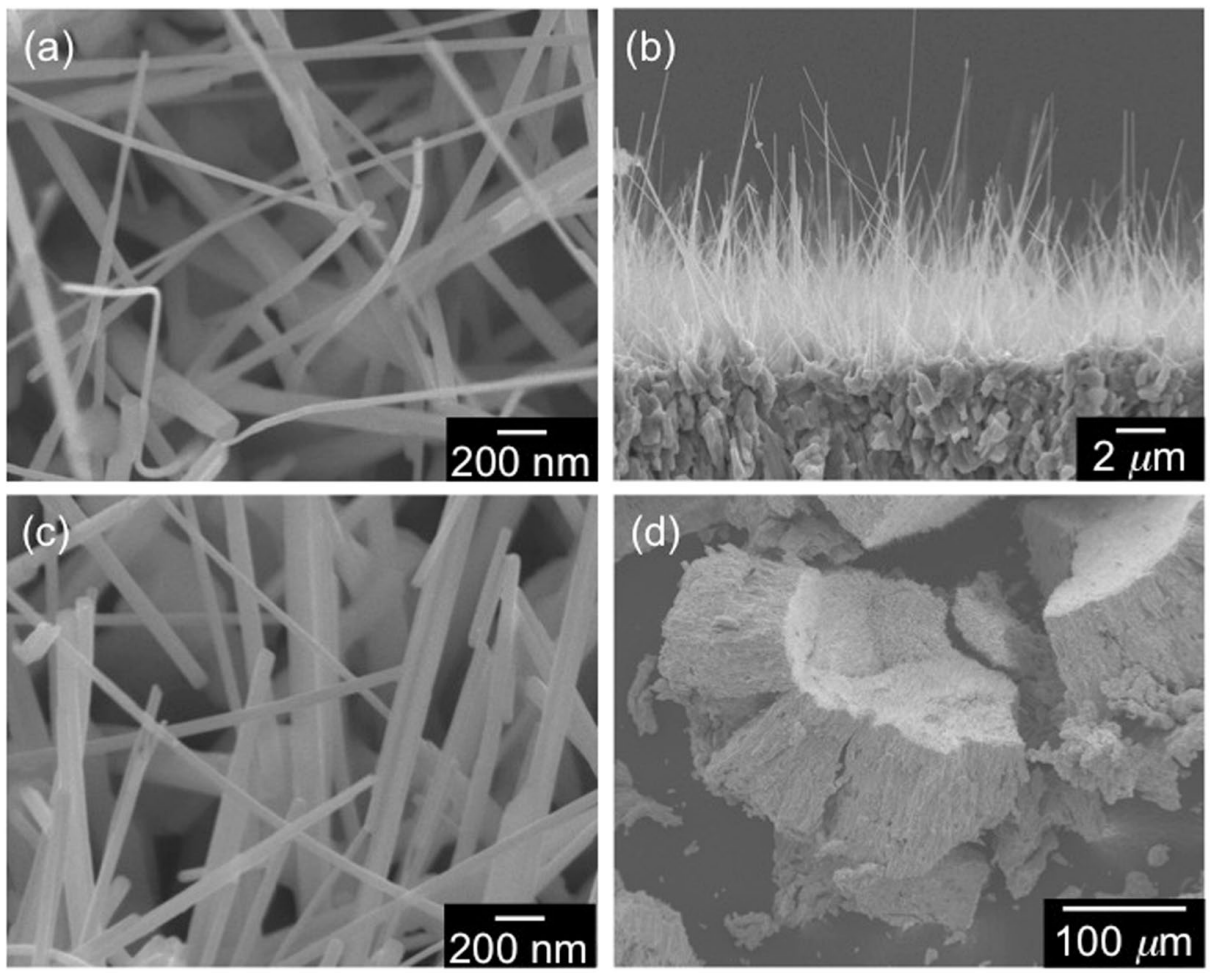
FE-SEM images of (**a**,**b**) Nb_2_O_5_:Rh-NW(F) and (**c**,**d**) Nb_2_O_5_:Rh-NW(P). (**a** and **c**) are the magnified views of (**b** and **d**), respectively.

Figure 2 shows the FE-SEM images of the nanowires synthesized from Nb foil with two different thicknesses, Nb_2_O_5_:Rh-NW(F) and Nb_2_O_5_:Rh-NW(P). Nb_2_O_5_:Rh-NW(F) forms nearly-vertical, free-standing nanowires with diameters ranging from 30 to 140 nm (Fig. 2a and b). Since the diameter dimension is similar to that of the undoped counterpart film [Nb_2_O_5_-NW(F), Figure S1], Rh(acac)(CO)_2_, which was constantly provided as a Rh dopant during the Nb_2_O_5_ synthesis, does not have a visual impact on the product morphology. The nanowires observed in Nb_2_O_5_:Rh-NW(P) possess similar diameters but are linked to the cornerstones with sizes of several µm (Fig. 2c and d).

The diffraction pattern of Nb_2_O_5_:Rh-NW(P) corresponds to the structure of *monoclinic* Nb_2_O_5_ (PDF 37-1468, Figure S2). XRD also indicates no existence of Rh_2_O_3_ that is envisioned to form by oxidative thermal decomposition of Rh(acac)(CO)_2_, suggesting one of two possibilities: a lack or a doping of Rh in the lattice. In principle, the latter should be accompanied by a peak shift depending on the magnitude of the doping metal ions incorporated into the lattice, as formulated by Bragg. In this case, however, the ionic radius of six-coordinated Nb^5+^ is between those of Rh^3+^ and Rh^4+^, both of which can stably exist in the metal oxide [ionic radii (Å); Nb^5+^:0.64, Rh^3+^:0.665, Rh^4+^:0.6). This means that, if Rh as a dopant possesses a mixed-valence state, it is hard to predict whether a peak shift, based on Bragg’s law, will occur.

The XPS spectrum of Nb_2_O_5_:Rh-NW(P) shows the clear presence of Rh; however, it was not easy to determine whether Rh introduced in the oxide nanowire possesses trivalent and/or tetravalent states, due to the small inter-peak distance (~0.3 eV, Figure S3)^35^. The molar ratio of Rh to Nb, determined from the spectral deconvolution, was 0.13.

**Figure 3:**
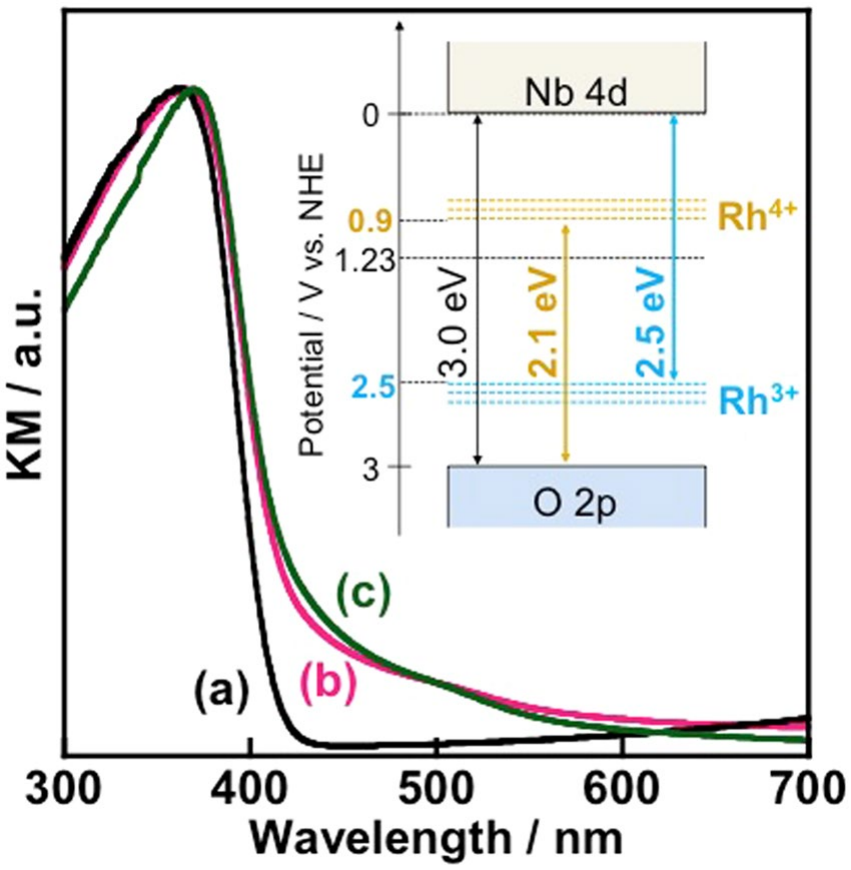
DRS of (**a**) Nb_2_O_5_-NW(P), (**b**) Nb_2_O_5_:Rh-NW(P), and (**c**) Nb_2_O_5_:Rh-NW(P) that was subjected to H_2_ at 673 K, followed by calcination at 1073 K in air. The latter process was carried out because the Rh dopant and the Nb host are reduced by the H_2_ treatment, resulting in a large increase in absorption due presumably to low-valent Nb, particularly at wavelengths longer than 500 nm. The energy gaps are calculated from the intersection of the tangential line from the given absorption and baseline. The inset shows the band structure constructed from the DRS results.

Nb_2_O_5_:Rh-NW(P) shows distinct visible absorption that is absent in the corresponding undoped counterpart, Nb_2_O_5_-NW(P) (Fig. 3). The emergent spectral shape largely differs from that of the Nb_2_O_5_/Rh_2_O_3_ mixture (Figure S4), whereas there was shown to be no major difference in those of Rh-doped TiO_2_ and SrTiO_3_^36, 37^. Nb_2_O_5_:Rh-NW(P) was then reduced by H_2_ to confirm the oxidation state of Rh. The absorptions at approximately 427 nm increases with the H_2_ reduction treatment, while the feature at 600 nm decreases. These observed phenomena are similar to those of SrTiO_3_:Rh with both Rh^3+^ and Rh^4+^, and hence, we conclude that the optical transitions in the visible region result from the coexistence of Rh^3+^ and Rh^4+^ in Nb_2_O_5_. Therefore, Rh doping led to no peak shift in the XRD pattern of Nb_2_O_5_ (Figure S2). The inset of Fig. 3 shows a predicted energy diagram of Nb_2_O_5_:Rh-NW, which is constructed based on earlier studies^36, 37^. The energy-gap values for Nb_2_O_5_:Rh-NW are as follows: the energy gaps between the valence-band maximum, formed mainly from O 2p orbitals, and the Rh^4+^ impurity level is 2.1 eV and between the Rh^3+^ impurity level and the conduction-band minimum, formed mainly from Nb 4d orbitals, is 2.5 eV.

### Plausible growth mechanism

The non-doped Nb_2_O_5_-NW(F) is proposed to grow through a self-catalytic growth mechanism^38^. The nanoparticles derived from Rh(acac)(CO)_2_, observed in the initial stages of the nanowire growth, are the last to disappear. This indicates that a successive supply of Rh does not hinder the nanowire growth and makes the penetration of Rh to the Nb_2_O_5_ crystal lattice easier. On the other hand, cornerstones sustaining nanowire bundles are found in Nb_2_O_5_:Rh-NW(P) (Fig. 2d). This observation results from a segregation phenomenon accompanied by the complete disappearance of metallic Nb, resulting in the remaining planar Nb_2_O_5_ substrate becoming more brittle than the substrate with the metallic Nb backbone.

### Photocatalytic performance

**Figure 4:**
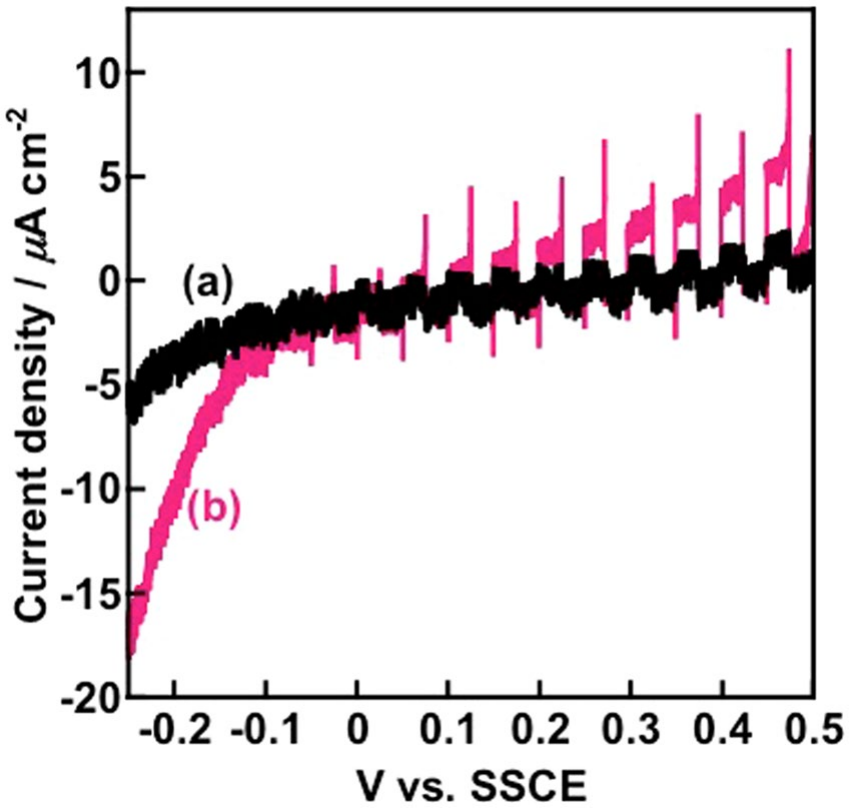
Current density vs. voltage characteristics of (**a**) Nb_2_O_5_-NW(F) and (**b**) Nb_2_O_5_:Rh-NW(F) taken with a sweep rate of 10 mV min^−1^ under irradiation of visible light from a 100-W Xe lamp with a Y44 cutoff filter. A 0.1 M phosphate buffer solution was used as an electrolyte.

The photoelectrochemical performances of Nb_2_O_5_:Rh-NW(F) and Nb_2_O_5_-NW(F) as a reference were examined under visible-light irradiation (*λ* > 440 nm). For the linear sweep voltammograms shown in Fig. 4, Nb_2_O_5_:Rh-NW(F) showed a well-marked, higher anodic current at potentials greater than 0.2 V (vs. SSCE) than Nb_2_O_5_-NW(F). The Faradaic efficiency at 0.45 V for the Nb_2_O_5_:Rh-NW(F) photoanode was estimated to be 99%, indicating that the observed photocurrent results from O_2_ evolution (Figure S5). On the other hand, the Y-44 filter used in the experiments does not possess light transmittance below 430 nm. Nb_2_O_5_-NW(F) showed an imperceptible photoresponse, which could represent an oxygen vacancy-induced photoresponse^39^ due to the poor oxygen supply in our vacuum-processing system during Nb_2_O_5_ growth. In fact, Nb_2_O_5_-NW is tinged pale blue, which may also suggest a gradual increase in absorption at longer wavelengths (Fig. 3a).

**Figure 5:**
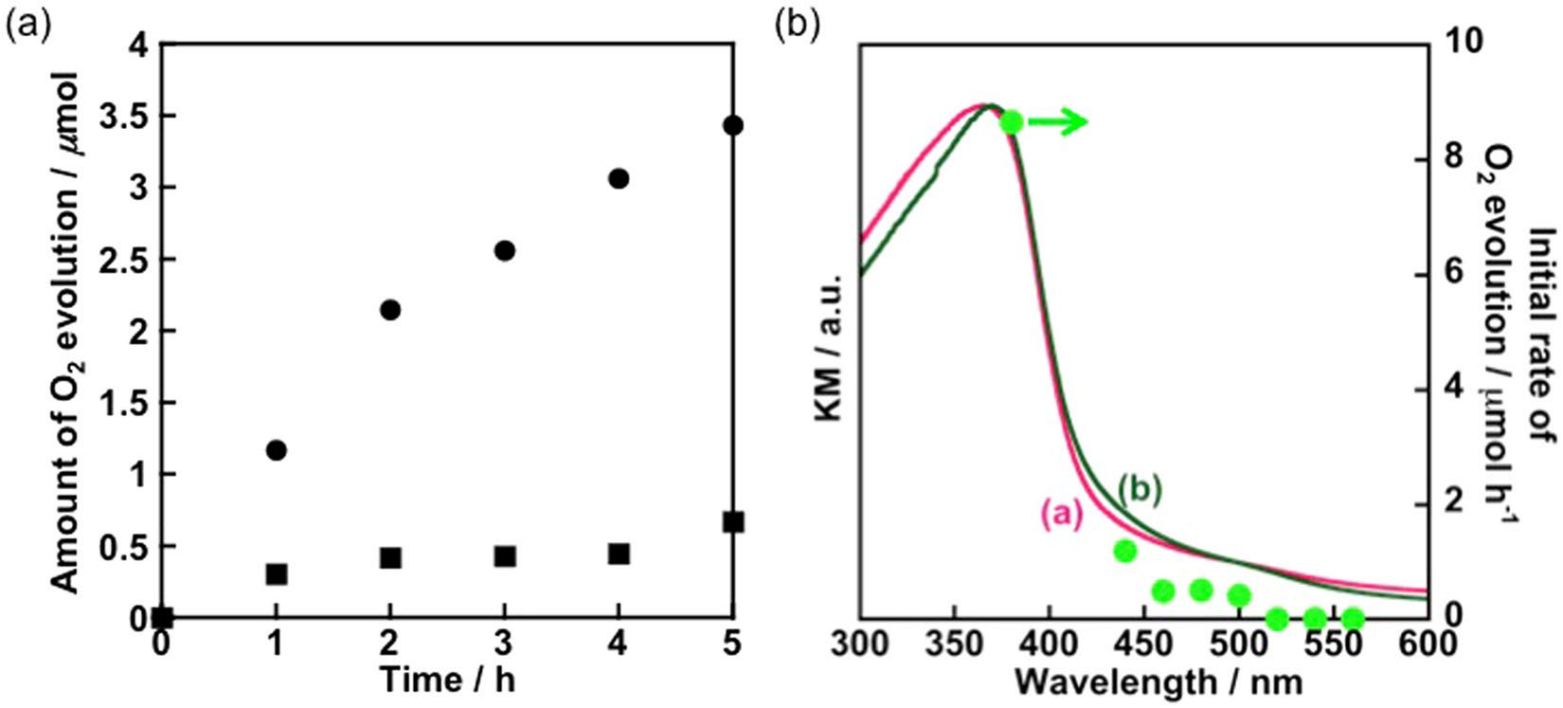
(**a**) Heterogeneous O_2_ evolution reactions over Nb_2_O_5_-NW(P) (black squares) and Nb_2_O_5_:Rh-NW(P) (black circles). Each sample weight is 0.3 g. A 0.01 mM aqueous AgNO_3_ solution and visible light provided from a 300-W Xe lamp with a Y44 filter were used as the sources of the electron acceptor and light, respectively. Figure 5b shows the wavelength dependence of the O_2_ evolution reaction (pale-green circles), together with the DRS spectra shown in Fig. 3.

Using the powdered counterpart, we examined the heterogeneous photocatalysis under identical irradiation conditions. Similar to the photoelectrochemical results, Nb_2_O_5_:Rh-NW(P) was found to oxidize water in the presence of an electron acceptor, Ag^+^, showing much higher activity than Nb_2_O_5_-NW(P) (Fig. 5a). In light of the energy structure, the observed oxygen evolution should be driven by the electronic transition from Rh^3+^ to the conduction band of the Nb_2_O_5_ host because electron transfer from the Rh^4+^ impurity level (supplied from the valence band of Nb_2_O_5_) to Ag^+^ is an endergonic process (Ag^+^  + e^−^ → Ag: *E* ^0^ = 0.8 V vs. NHE).

The wavelength dependence of the O_2_ evolution reaction, shown in Fig. 5b, clearly shows that the absorption edge from Rh^3+^ correlates well with the onset wavelength for O_2_ evolution. On the other hand, due to a lack of driving force for electron transfer from the conduction-band minimum of the host for water reduction (as shown in the band structure in Fig. 3), Nb_2_O_5_:Rh-NW(P) shows negligible photoresponse for H_2_ evolution, even with fine Pt particles deposited by a unique, intermittent irradiation of visible light (Figures S6 and S7).

### Investigating a heterostructure for efficient charge transfer

The combination of the metal oxide and sulphide is well suited to perform water splitting, as reported in recent studies^21–31, 40, 41^. Ideally, an H_2_-evolving sulphide, such as CdS, is placed on the reduction site of the O_2_-evolving oxide; however, the strategy is poorly known. The VLS mechanism, guided by a metal catalyst, has been extensively used for the controlled growth of nanomaterials. However, in general, the low permeability of the reactive gas into the powder hinders the reaction of the gaseous sulphide and Au on Nb_2_O_5_:Rh-NW(P). Therefore, a CVD reaction was carried out using mechanical rotation to help expose the unreacted powder to the gaseous sulphide^34^.

**Figure 6:**
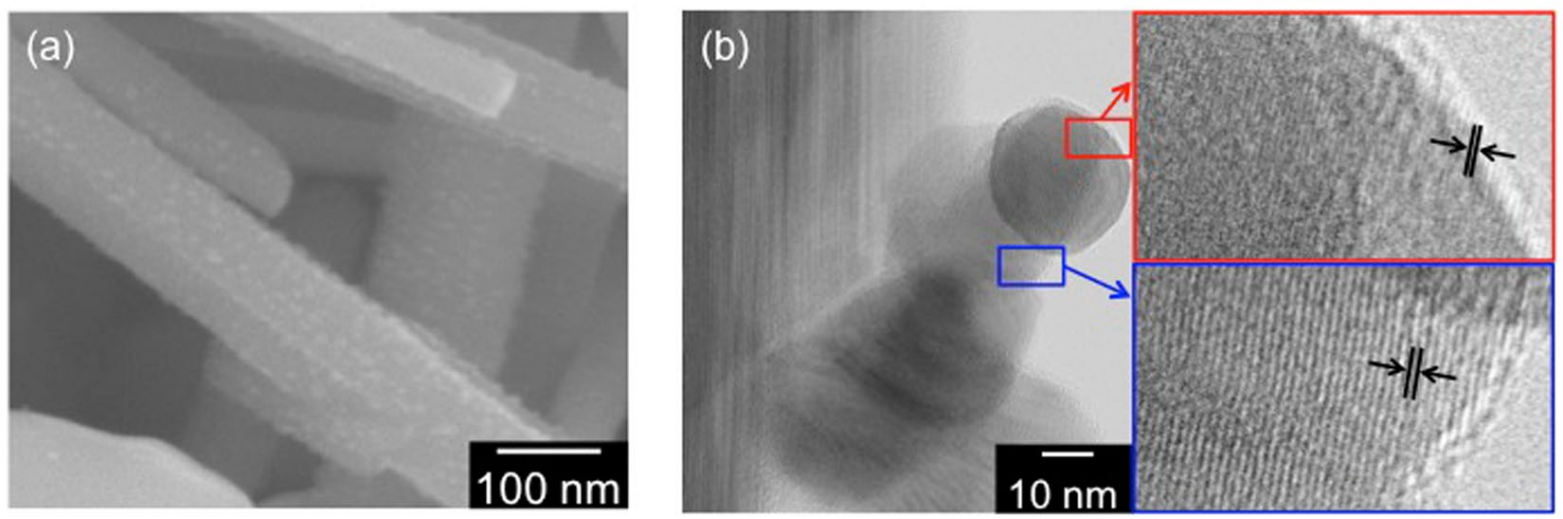
(**a**) FE-SEM image of Au/CdS/Nb_2_O_5_:Rh-NW(P) obtained from a short-term, rotating CVD process. (**b**) TEM images of Au/CdS/Nb_2_O_5_:Rh-NW(P). The calculated lattice spacings in the red and blue frames (2.1 and 3.1 Å) are in good agreement with the *d*-spacings of the (200) plane of Au (*d*_200_ = 2.039 Å) and of the (101) plane of CdS (*d*_101_ = 3.164 Å), respectively.

First, a Au nanocatalyst for the VLS mechanism was photochemically deposited on the reduction sites of Nb_2_O_5_:Rh-NW(P) in the presence of an electron donor [Au/Nb_2_O_5_:Rh-NW(P)]^19^. The existence of Au, with an undetectable size at the sensitivity of FE-SEM, on Nb_2_O_5_:Rh-NW(P) was confirmed from the Au 4f XPS spectrum (Figure S8). Upon prolonged a rotating CVD process with CdS, the nanostructure undergoes a change in shape to branched nanowires (Figure S9). Each branch possesses particulate tip, evidencing that the VLS mechanism occurred on the nanowire. The Raman spectra reveal the growth of peaks corresponding to CdS, indicating that the branches are composed of CdS (Figure S10). Considering the carrier-transfer distance, a shorter branch should be favourable for electron transfer across the oxide-sulphide interface. Figure 6a shows an FE-SEM image indicating that a short-term, rotating CVD process results in highly dispersed nanoparticles with an average size of 17 nm throughout the nanowires [Au/CdS/Nb_2_O_5_:Rh-NW(P)]. Additionally, TEM images show the existence of CdS with a Au tip on a nanowire (Fig. 6b).

**Figure 7:**
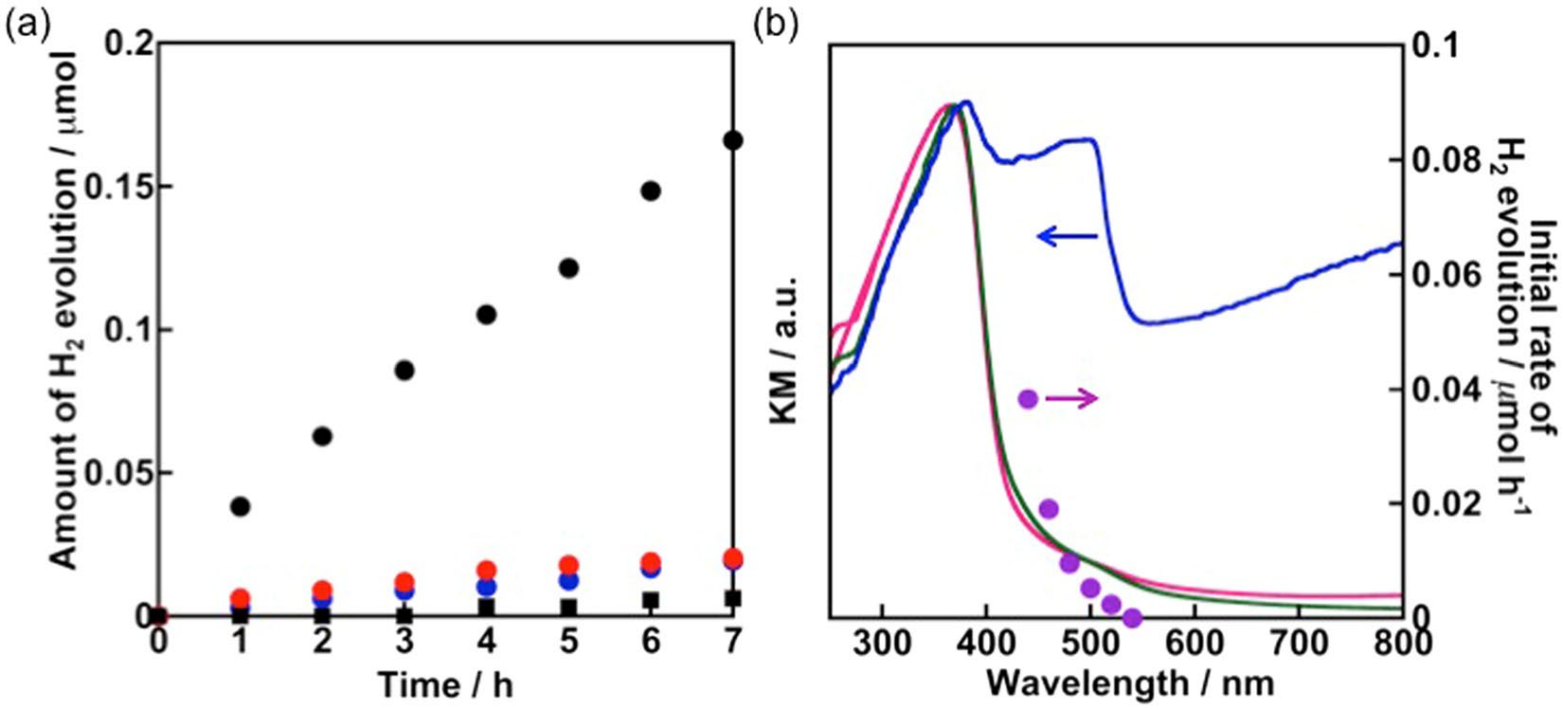
(**a**) Heterogeneous H_2_ evolution over Au/CdS/Nb_2_O_5_:Rh-NW(P) (black circles), Nb_2_O_5_:Rh-NW(P) with randomly deposited CdS (red circles), CdS (blue circles), and Au/Nb_2_O_5_:Rh-NW (P) (black squares). Each sample weight is 0.1 g, except for CdS (0.31 mg). A 10 vol% aqueous methanol solution and visible light provided from a 300-W Xe lamp with a Y44 filter were used as the sources of the electron donor and light, respectively. Figure 7b shows the wavelength dependence for H_2_ evolution over Au/CdS/Nb_2_O_5_:Rh-NW(P) (purple circles), together with the DRS of Au/CdS/Nb_2_O_5_:Rh-NW(P) (blue line) and Nb_2_O_5_:Rh-NW(P) (red and green lines, as shown in Fig. 3).

Under irradiation of visible light (*λ* > 440 nm) in a 10 vol% methanol solution, Au/CdS/Nb_2_O_5_:Rh-NW(P) achieves an order of magnitude higher performance for H_2_ evolution than the following standards: Pt/Nb_2_O_5_:Rh-NW(P), Au/Nb_2_O_5_:Rh-NW(P), an equivalent amount of CdS contained in Au/CdS/Nb_2_O_5_:Rh-NW(P), and Nb_2_O_5_:Rh-NW(P) with randomly deposited CdS (Fig. 7a). In addition, the wavelength dependence, shown in Fig. 7b, clearly indicates that CdS loaded onto the nanowires does not perform well by itself (shown with the blue circles in Fig. 7a) at 520 nm, corresponding to the absorption by only CdS. When a shorter wavelength is utilizing corresponding to the absorption edge of Nb_2_O_5_:Rh-NW, a steep increase in H_2_ evolution activity is observed. Therefore, the observed photocatalytic H_2_ evolution over Au/CdS/Nb_2_O_5_:Rh-NW(P) is driven by electron transfer from two-photon excitation. The rate of H_2_ evolution decreased during 11.5 h of photocatalytic reaction (Figure S11a); however, the elution of CdS was not seen in ICP experiments. On the other hand, the nanowire structures were found to partially decompose possibly by stirring in a reaction cell, which can be sometimes seen with other nanowire system (Figure S11b). Au is frequently used as a cocatalyst for H_2_ evolution and as a photosensitizer to harness its surface plasmon resonance^42, 43^. In this work, the loaded Au provided a modest effect on H_2_ evolution over Nb_2_O_5_:Rh-NW(P) (Fig. 7a). The degree of improvement will be not significant, even over CdS, particularly considering previous research^44^.

**Figure 8:**
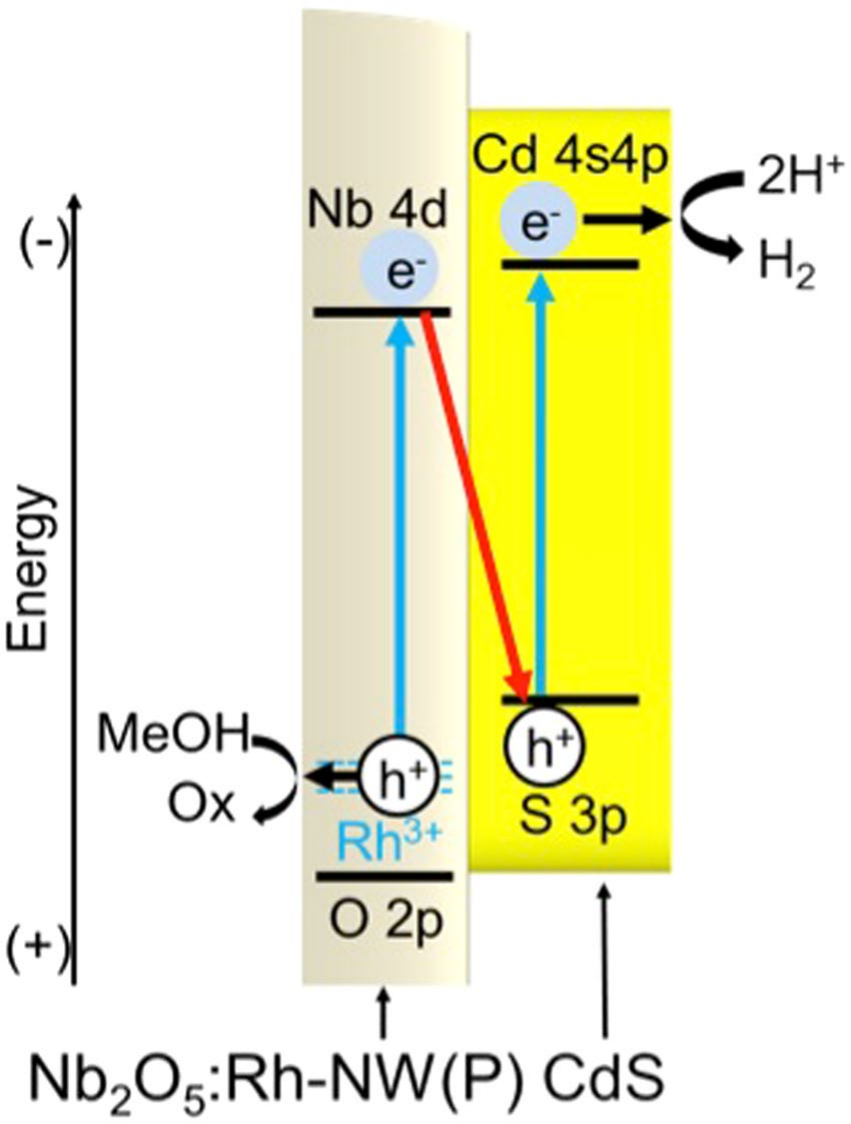
Plausible primary electron-transfer mechanism occurring on Au/CdS/Nb_2_O_5_:Rh-NW(P).

In the light of these results, a plausible primary reaction mechanism is shown in Fig. 8. Following visible-light capture, an electron-hole pair is generated in both Nb_2_O_5_:Rh-NW and CdS. The hole in the Rh^3+^ impurity level is consumed by methanol, and the electron in the conduction band of CdS reduces water to form H_2_. In addition to methanol^45^, the conduction-band electron in Nb_2_O_5_:Rh-NW promptly merges with the valence-band hole in CdS due to a larger driving force for electron transfer than the driving force for water reduction. If electron transfer follows the heterojunction^46^, photogenerated electrons accumulate on the conduction band of Nb_2_O_5_:Rh-NW, and holes accumulate on the valence band of CdS. In that case, the photocatalyst composite will show no photoresponse, as with bare Nb_2_O_5_:Rh-NW(P), because Au is located on top of CdS. However, H_2_ evolution was highly improved, indicating that H_2_ is evolved over CdS rather than Nb_2_O_5_:Rh-NW. Furthermore, hole accumulation should facilitate photocorrosion of CdS; however, such a phenomenon was not observed. Thus, we conclude that electron transfer mainly follows a Z-scheme. The vast improvement originates from CdS directly linking to the reduction site of Nb_2_O_5_:Rh-NW and hence efficient electron transfer across the oxide-sulphide nanointerface occurs.

## Conclusions

Self-catalytic growth and MOCVD were combined in a single synthesis approach to form Nb_2_O_5_:Rh-NW with two different macroscopic morphologies. Rh was doped into the Nb_2_O_5_ lattice, forming mixed-valence Rh^3+^ and Rh^4+^ states. The free-standing nanowire film functioned as a photoanode for photoelectrochemical water splitting under visible-light irradiation (*λ* > 440 nm). The powder was found to oxidize water in the presence of an electron acceptor. To boost the H_2_-evolution performance, Au was photodeposited on the reduction site of Nb_2_O_5_:Rh-NW, and using a rotating CVD apparatus, CdS was directly bonded to the reduction site through a VLS mechanism. The 3D heteronanostructure consisting of Nb_2_O_5_:Rh-NW stems with short Au/CdS branches showed largely improved H_2_-evolution performance due to efficient carrier transfer at the oxide-sulphide nanointerface.

## Methods

0.1 and 0.25 mm-thick Nb foils (99.9%) were purchased from the Nilaco Corporation. Rh(acac)(CO)_2_ (acac = acetylacetonato, 39.9% as Rh, Tanaka Kikinzoku Kogyo) was employed as Rh source in the MOCVD process. CdS was purchased from Aldrich (99.995%). About other chemical substances we used those of high-grade qualities.

### Nb_2_O_5_ nanowire fabrication with and without Rh doping

The film materials, Nb_2_O_5_-NW(F) and Nb_2_O_5_:Rh-NW(F), and the powdered forms, Nb_2_O_5_-NW(P) and Nb_2_O_5_:Rh-NW(P), were synthesized from 0.1- and 0.25-mm-thick Nb foils, respectively. Prior to the synthesis, Nb foil with dimensions of 1 cm × 2 cm was rinsed with water and then ethanol three times under sonication for 3 min. The foil was finally rinsed in acetone and dried under a N_2_ flow. The clean foil was placed at the centre of a quartz tube reactor [inner diameter (i.d.) = 1.6 cm] equipped with an electric furnace with a length of 14 cm. The following modified Varghese’s method was used to obtain the non-doped nanowires, Nb_2_O_5_-NW(F) and Nb_2_O_5_-NW(P)^37^. After the pressure in the quartz tube reached ~15 Pa, the sample tube was heated to 1173 K with a ramping rate of 318 K min^−1^ while flowing 100-sccm Ar through an 1/8-inch stainless steel tubing (reaching a total pressure of ~1.2 × 10^3^ Pa). O_2_ (400 sccm) was introduced through the same-sized tubing for 1.5 h. Rh-doped nanowires, Nb_2_O_5_:Rh-NW(F) and Nb_2_O_5_:Rh-NW(P) were grown under the same conditions, with the exception that Ar containing Rh(acac)(CO)_2_, vaporized at 343 K, and O_2_ were utilized at the same time.

### Fabrication of the Rh-doped Nb_2_O_5_ nanowires with CdS

Using a photodeposition method, a Au catalyst was loaded on reduction sites of Nb_2_O_5_:Rh-NW(P) (Au/Nb_2_O_5_:Rh-NW). Nb_2_O_5_:Rh-NW(P) was mixed in a 10 vol% aqueous methanol solution of HAuCl_3_ (42 μmol L^−1^), and the suspension stirred at 400 rpm was subjected to 20 cycles of a 10-s irradiation of visible light (*λ* > 440 nm). The intermittent irradiation allowed the Au catalyst to deposit with a tiny size, as observed in Figure S8. We used a home-built CVD apparatus with a double-walled quartz tube, inside of which housed two different powders, rotating independently by a rotary manipulator (ARIOS, RFT34S) and possessing a hole leading towards external evacuation [i.d.s of the inner and outer tubes were 1.6 and 0.5 cm, respectively]. CdS and Au/Nb_2_O_5_:Rh-NW were placed at the centre and at the downstream end of the furnace, respectively. After evacuation (~15 Pa), the quartz tube was heated to 1073 K for 40 min while flowing 25-sccm Ar and rotating at a speed of 30 rpm, resulting in the formation of CdS/Au/Nb_2_O_5_:Rh-NW. As a reference material, CdS [0.31 mg, equivalent to the amount of CdS contained in Au/CdS/Nb_2_O_5_:Rh-NW(P)] was randomly deposited on top of Nb_2_O_5_:Rh-NW(P) (100 mg) by an impregnation method. The CdS loading was evaluated by processing CdS/Au/Nb_2_O_5_:Rh-NW in HCl and analysing the eluent by ICP-AES.

### Photocatalytic experiments

A standard three-electrode measurement combining the working electrode, a Pt coil counter electrode, and an Ag/AgCl (SSCE) reference electrode was carried out in a 0.1 M phosphate buffer solution to assess the photoelectrochemical performance. A 100-W Xe lamp (USIO, UXL-500SX) with a Y44 filter was used as the light source. Heterogeneous H_2_ and O_2_ evolution reactions were examined using a gas-closed circulation system equipped with GC (Shimadzu, GC-8A). The photocatalyst was dispersed in a stirred aqueous solution (120 mL, 400 rpm), containing an electron donor (methanol) or an acceptor (AgNO_3_), and was irradiated with visible light from 300 W Xe-lamp (CERMAX^®^, PE300BF) with a Y44 cut-off filter. For the experiments regarding wavelength dependence, incident-light-wavelengths were controlled by varying cut-off filters.

### Material characterizations

Followings are the facilities used for characterizations of materials obtained. XRD (Rigaku MiniFlex600, Cu *Kα*), FE-SEM (JEOL JSM-6500F), DRS (JASCO V-670), Raman (Horiba LabRAM HR-800, excitation wavelength of 532 nm), XPS (JEOL JPS-9000, Mg K*α* as an X-ray source), and ICP-AES (Seiko Instrument Inc. SPS1500).

### Data availability

All data are included in this published article (and its Supplementary Information files).

## Electronic supplementary material

Revised supporting information
