# Supplementary material for: Morphology Control of Energy-Gap-Engineered Nb2O5 Nanowires and the Regioselective Growth of CdS for Efficient Carrier Transfer Across an Oxide-Sulphide Nanointerface
Source: Sci Rep. 2017 Jul 7;7:4913. doi: 10.1038/s41598-017-05292-2 (PMC5501808; doi:10.1038/s41598-017-05292-2)
Supplement: Supplementary file 1 — Revised supporting information [file 41598_2017_5292_MOESM1_ESM.pdf]

Supporting Information

Morphology Control of Energy-Gap-Engineered Nb_2O_5 Nanowires and the Regioselective Growth of CdS for Efficient Carrier Transfer Across an Oxide-Sulphide Nanointerface

*Tomoki Shinohara, Miyu Yamada, Yuki Sato, Shohei Okuyama, Tatsuto Yui, Masayuki Yagi, Kenji Saito**

Department of Materials Science and Technology, Faculty of Engineering, Niigata University, 8050 Ikarashi-2, Niigata 950-2181, Japan

Correspondence to ksaito@eng.niigata-u.ac.jp

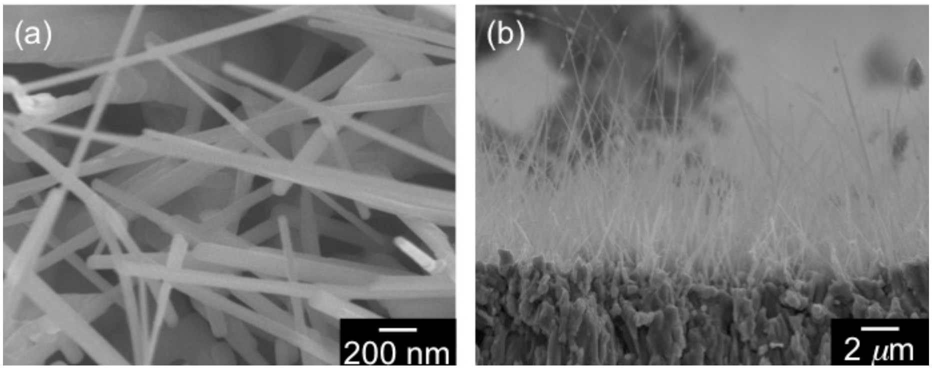

Figure S1. FE-SEM image of Nb_2O_5-NW(F), taken from the (a) top and (b) side surfaces, respectively.

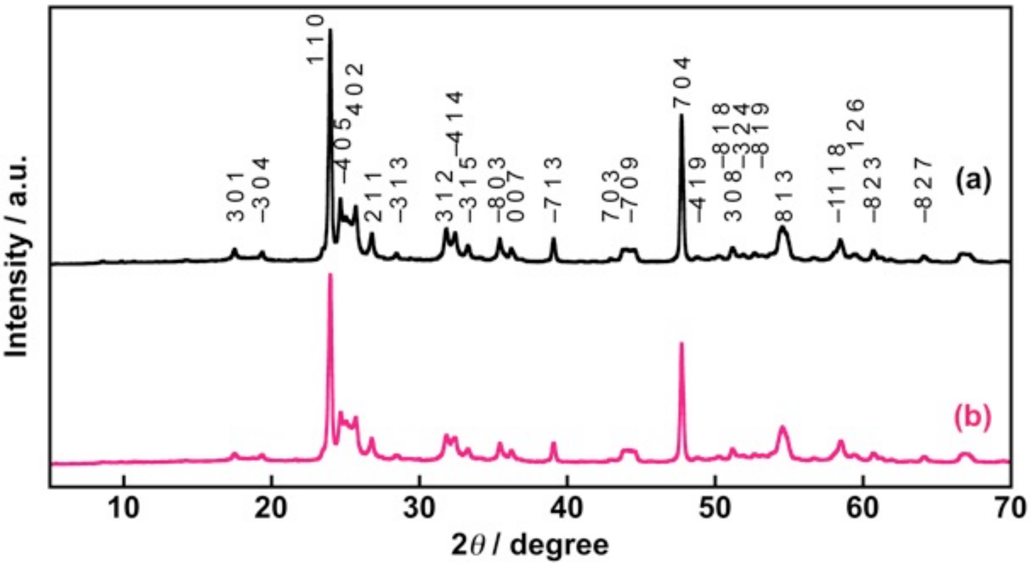

Figure S2. XRD patterns of (a) Nb_2O_5-NW(P) and (b) Nb_2O_5:Rh-NW(P).

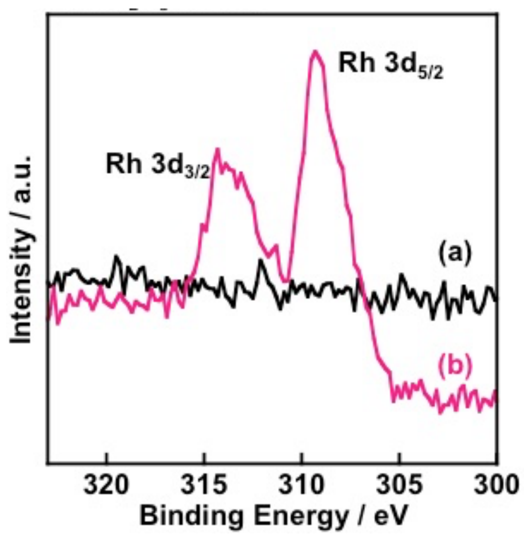

Figure S3. XPS spectra of (a) Nb_2O_5-NW(P) and (b) Nb_2O_5:Rh-NW(P). Binding energies were calibrated with respect to the C1s value of contaminated carbon (284.6 eV).

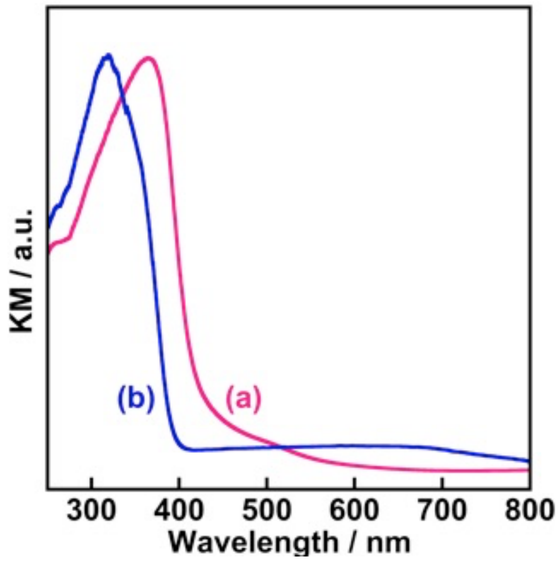

Figure S4. DRS of (a) Nb_2O_5:Rh-NW(P) (the same spectrum shown in Figure 3b) and the Nb_2O_5/Rh_2O_3 mixture with a composition corresponding to the Nb_2O_5:Rh-NW(P).

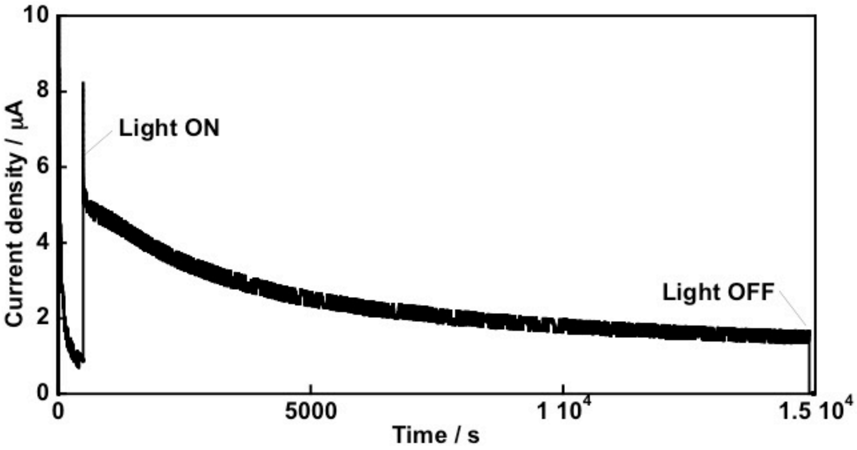

Figure S5. A chronoamperometry experiment using Nb_2O_5:Rh-NW(F) (electrode area: 0.54 cm^2). A 0.1 mol L^{-1} phosphate buffer solution and visible light from 100 W Xe lamp with an Y44 cutoff filter were used as an electrolyte and light source, respectively.

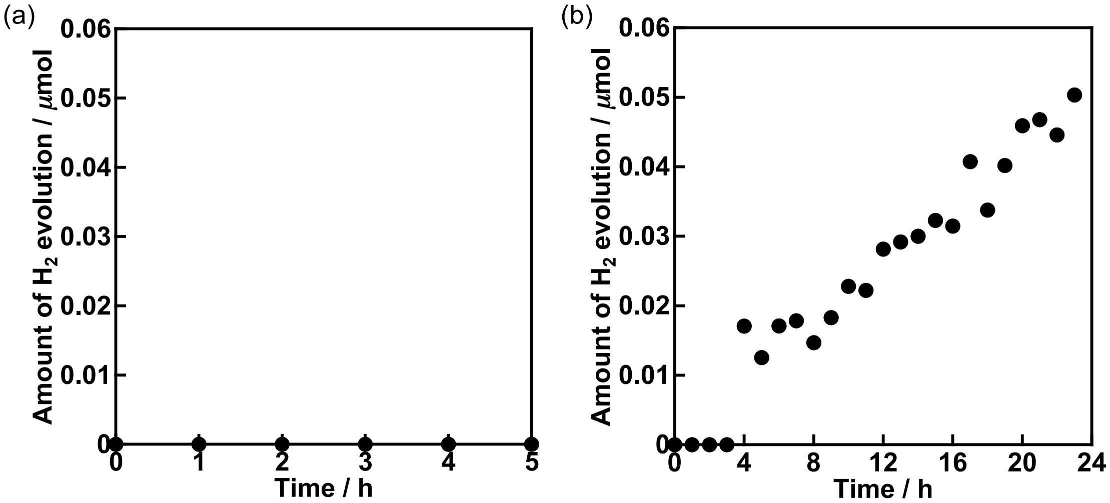

Figure S6. Heterogeneous H_2 evolution reactions over Nb_2O_5:Rh-NW(P) with Pt cocatalyst (sample weight: 0.3 g) that was deposited by (a) ambient or (b) intermittent light. 10 vol% of an aqueous methanol solution and visible light provided from 300 W Xe lamp with an Y44 filter were used as the sources of electron donor and light, respectively.

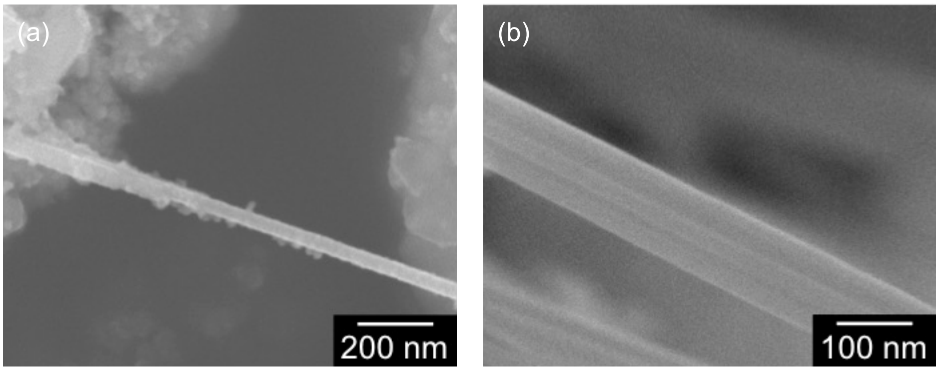

Figure S7. FE-SEM images of Nb_2O_5:Rh-NW(P) with 1wt% of Pt cocatalyst that was deposited by (a) ambient light. Using identical precursor solution, Pt was deposited from intermittent light (b).

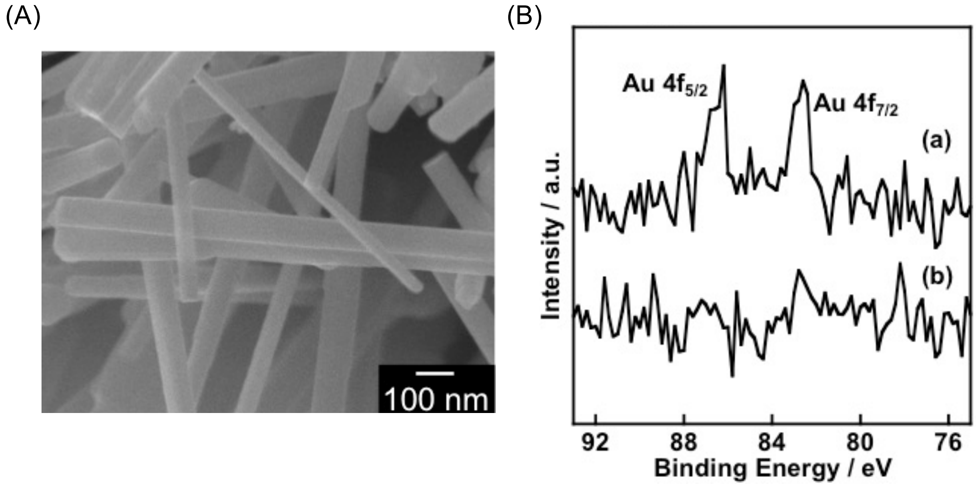

Figure S8. (A) FE-SEM image and (B) (a) Au 4f XPS spectra of Au/Nb_2O_5:Rh-NW(P). (b) in Figure B is taken from Nb_2O_5-NW(P) as the reference. Binding energies were calibrated with respect to the C1s value of contaminated carbon (284.6 eV).

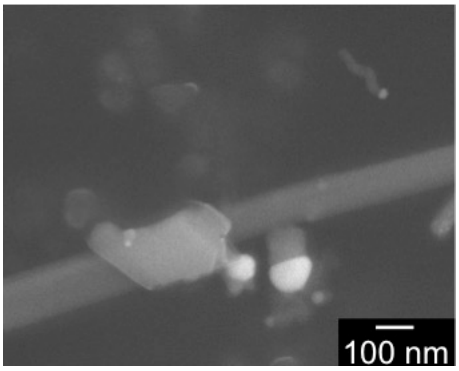

Figure S9. FE-SEM image showing CdS branch with Au tip on the Nb_2O_5:Rh-NW(P) stem.

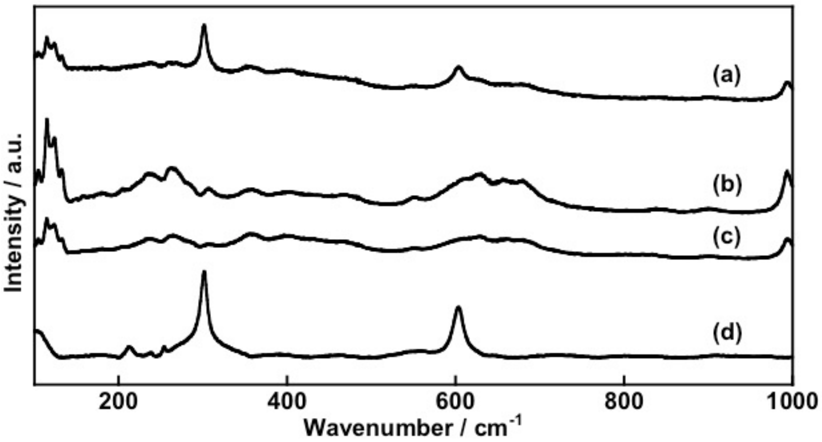

Figure S10. Raman spectra of (a) Au/CdS/Nb_2O_5:Rh-NW(P), (b) Au/Nb_2O_5:Rh-NW(P), (c) Nb_2O_5:Rh-NW(P), and (d) CdS.

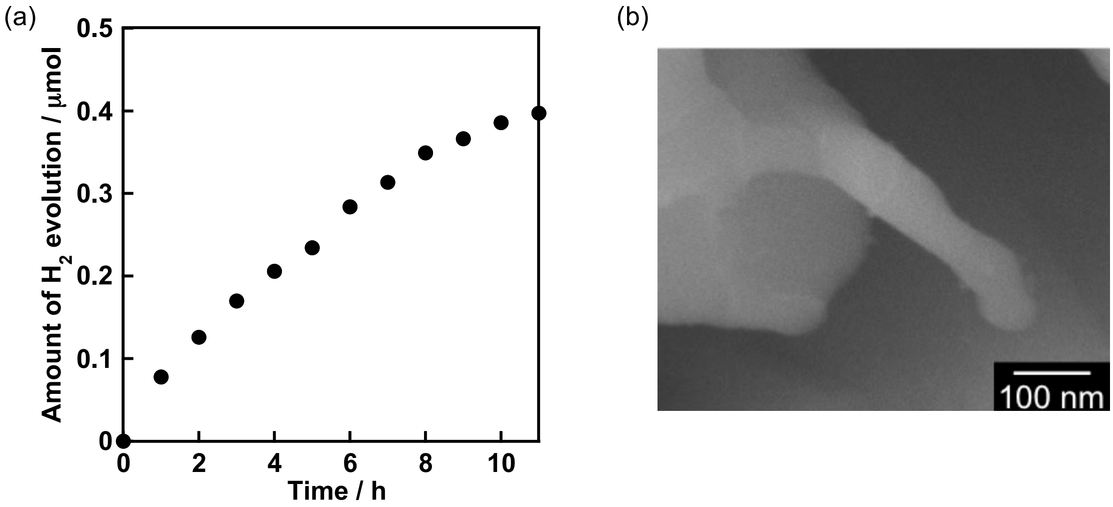

Figure S11. (a) Long-term heterogeneous H_2 evolution reaction over Au/CdS/Nb_2O_5:Rh-NW (P). 10 vol% of an aqueous methanol solution and visible light provided from 300 W Xe lamp with an Y44 filter were used as the sources of electron donor and light, respectively. (b) shows FE-SEM image of the photocatalyst powder after the reaction.
